
JOURNAL INFORMATION
==============================
NLM Title Abbreviation: Atlanta J Rec Med
NLM Journal ID: 0001011

Atlanta Journal-Record of Medicine

Publisher: Biomedical Journal Digitization Project

ARTICLE INFORMATION
==============================
PMCID: PMC9046035
PMID: 36020482
Article version: 1
Subjects: Medical Items

Medical Items

Print publication date: 1909 Feb
Volume: 11
Issue: 11
First page: 613
Last page: 614
License: This work is free of known copyright restrictions. For more information, please see PMC Back Issue Digitization.
License URL: https://creativecommons.org/publicdomain/mark/1.0/

==============================

### A Palatable Food-Fat for Children

**Some children dislike the taste of cod-liver oil.**

But it's the exceptional child **that won't take** Hydroleine and ask for more.

It has a smooth, delicate, nutty flavor that neither offends nor tires the palate — a matter of importance when the use of the oil is to be long continued.

Hydroleine is simply pure, fresh cod-liver oil perfectly emulsified and rendered palatable, stable and — unusually digestible. Sold by druggists.

Sample with literature on request

THE CHARLES N. CRITTENTON CO.
115 FULTON ST., NEW YORK

### MEDICAL ITEMS

### PHYSIOLOGICAL CELL NUTRIFICATION.

In this impelling period of intense activities the vital forces are often called upon to appropriate their substance to the limit of structural stamnia. The consuming folly of unbridaled ambition now prevailing depletes the organism, impoverishing the cellular structures of their protoplasmic content and establishing a condition of metabolic retrogression and a faulty and embarrasses neurogenesis.

To meet these conditions the most pressing therapeutic needs is for efficient reconstructants to the neuroblastic process.

That neurasthinic and anemic manifestations and propensities are increasing to a disquieting extent is evident, and to provide physiological and reconstructive agents to encourage the process of cellular co-ordination and progression i sthe considerable labor in which the biological and pharmaceutical chemist is now most engaged.

That basic nutritional therapy directed to the physiological processes of cell genesis is of paramount importance is shown by the growing interest of the physiologist and the physician in this comprehensive avenue of applied therapeutics.

It has been shown that iron, manganese and phosphorus constitute in the human economy the salient material elements most essential to tissue elaboration and prpopgrpession.

Iron, if in a form suitable for sell absorption, enriches the hemaglobin content of the blood and contributively effects, with the manganese, a healthy and normal arterialization. According to Loomis, Bunge and others, maganese exerts on oxigenating influence in excess of iron; the two elements together, however, excite a vigorous stimulation of the sluggish arterial currents, encouraging a return to progressive and sustained cell genesis.

That organic iron and manganese have, in therapeutics, established their ascendenc over allied inorganic substances is not remarkable when it is remembered that each of these elements is found to exist in the economy in the form of an albuminate.

Notwithstanding these facts there has been some disappointnment in the use of physilogical iron and manganese. This partial failure may be traced to a very logical circumstance—a conclusion proved beyond the postulate.

Clearly, in the light of biological research, iron and manganese, while important and useful as blood oxigenators and enrichers, do not fully represent the correlation of reciprocal elements esesntial to a vigorous and sustained hematosis; nor are they sufficient in themselves to establish progressive tissue elaboration; physiological phosphorus is required to round out the therapy of these valuable hematinics.

It was to meet the growing demand for a true physiological phosphoid nutrient and tissue builder that Phospho-Ferrum was offered the clinical body.

This preparation represents organic iron, organic manganese, phpypsiological phosphorus—the lime salt of glycerophosphoric acid—and beef proteids, compounded into an agreeable liquid galenical of a deep vinous color and a grateful, aromatic odor and taste.

Phospho-Ferrum has been fully tried; it is now on a firm footing and is supplied by all progressive pharmacists.

—
